# Protection Motivation Perspective Regarding the Use of COVID-19 Mobile Tracing Apps Among Public Users: Empirical Study

**DOI:** 10.2196/36608

**Published:** 2023-03-01

**Authors:** Pamella Howell, Mohamed Abdelhamid

**Affiliations:** 1 Department of Information Systems California State University Los Angeles Los Angeles, CA United States; 2 Department of Information Systems California State University Long Beach Long Beach, CA United States

**Keywords:** COVID-19, mobile tracing app, protection motivation theory, privacy concerns, global health

## Abstract

**Background:**

Access to data is crucial for decision-making; this fact has become more evident during the pandemic. Data collected using mobile apps can positively influence diagnosis and treatment, the supply chain, and the staffing resources of health care facilities. Developers and health care professionals have worked to create apps that can track a person’s COVID-19 status. For example, these apps can monitor positive COVID-19 test results and vaccination status. Regrettably, people may be concerned about sharing their data with government or private sector organizations that are developing apps. Understanding user perceptions is essential; without substantial user adoption and the use of mobile tracing apps, benefits cannot be achieved.

**Objective:**

This study aimed to assess the factors that positively and negatively affect the use of COVID-19 tracing apps by examining individuals’ perceptions about sharing data on mobile apps, such as testing regularity, infection, and immunization status.

**Methods:**

The hypothesized research model was tested using a cross-sectional survey instrument. The survey contained 5 reflective constructs and 4 control variables selected after reviewing the literature and interviewing health care professionals. A digital copy of the survey was created using Qualtrics. After receiving approval, data were collected from 367 participants through Amazon Mechanical Turk (MTurk). Participants of any gender who were 18 years or older were considered for inclusion to complete the anonymized survey. We then analyzed the theoretical model using structural equation modeling.

**Results:**

After analyzing the quality of responses, 325 participants were included. Of these 325 participants, 216 (66.5%) were male and 109 (33.5%) were female. Among the participants in the final data set, 72.6% (236/325) were employed. The results of structural equation modeling showed that perceived vulnerability (*β*=0.688; *P*<.001), self-efficacy (*β*=0.292; *P*<.001), and an individual’s prior infection with COVID-19 (*β*=0.194; *P*=.002) had statistically significant positive impacts on the intention to use mobile tracing apps. Privacy concerns (*β*=−0.360; *P*<.001), risk aversion (*β*=−0.150; *P*=.09), and a family member’s prior infection with COVID-19 (*β*=−0.139; *P*=.02) had statistically significant negative influences on a person’s intention to use mobile tracing apps.

**Conclusions:**

This study illustrates that various user perceptions affect whether individuals use COVID-19 tracing apps. By working collaboratively on legislation and the messaging provided to potential users before releasing an app, developers, health care professionals, and policymakers can improve the use of tracking apps. Health care professionals need to emphasize disease vulnerability to motivate people to use mobile tracing apps, which can help reduce the spread of viruses and diseases. In addition, more work is needed at the policy-making level to protect the privacy of users, which in return can increase user engagement.

## Introduction

### Background

The BA.5, Omicron, and Delta variants of COVID-19 continue to fuel the global health crisis [1]. Federal, state, and local agencies, and many private sector organizations have taken steps to move the United States to the “new normal.” People often report positive COVID-19 test results and vaccination status to achieve this normalcy. For example, the Occupational Safety and Health Administration (OSHA) adopted a Healthcare Emergency Temporary Standard compelling all health care workers to be vaccinated or submit to frequent testing [2]. The Biden administration signed an executive order requiring COVID-19 testing and vaccination reporting for federal employees and companies receiving federal funding [3]. Major cities like Los Angles and New York also have mandates to report vaccination status for attending classes in grades K-12 and universities, indoor dining, or making purchases in shopping malls [4,5]. However, there is a general distrust about sharing COVID-19–related information, which has motivated personal and political legal challenges to reporting mandates [6].

This study examined individuals’ perceptions about sharing data on COVID-19–related metrics, such as testing frequency, diagnosis, and vaccination status, on mobile apps. Technologies like mobile apps are used to improve outcomes, increase patient participation in their care [7], and reduce the strain on limited health care resources. Further, since treatment regimens are developed slowly, information gathered and disseminated using mobile apps can be pivotal for stemming the spread of the disease. The use of COVID-19 tracing apps can also provide the public, businesses, and health care professionals with data to make informed decisions about the risks of infection. The findings of our study will fill an important gap in the literature, considering that more technologies are being created to mitigate the spread of COVID-19. A systematic review of COVID-19 mobile apps indicated that leveraging technology can be vital for combating the disease [8]; however, researchers have not examined the salient factors that support their use.

Using a modified version of the protection motivation theory (PMT), we explored individuals’ privacy concerns, risk aversion, COVID-19 infection status, vulnerability, and self-efficacy perception on their likelihood to use COVID-19 tracing apps. This research is one of the first to apply the PMT in the COVID-19 data–sharing context. Our theoretical contribution is 2-fold. First, we adapted the PMT by operationalizing response costs as privacy concerns. Second, we adapted threat appraisal by examining risk aversion and assessing an individual’s or their family members’ COVID-19 infection status. The rest of the paper is organized as follows: background literature on mobile apps used to fight COVID-19 and the PMT hypotheses, followed by a summary of the data collection and measurement model in the Methods section. The discussion and conclusion are in the final section.

### Mobile Technology as an Intervention Tool for Combating COVID-19

Early studies reviewing the use of mobile apps in health care showed promise. Now that mobile apps have been used for over a decade in health care, they are increasingly seen as necessary tools to promote evidence-based medicine. To achieve that aim, apps are being developed to educate patients and help health care professionals treat and diagnose various diseases [7]. Although mobile apps represent a small percentage of health care technology developed for use, they are the primary interface for Internet of Things (IoT) devices designed to improve patient experiences, reduce costs, and improve outcomes [9]. Improvements in IoT have contributed to the possibility of rapidly deploying health apps in a crisis like COVID-19 [10,11]. The expansion of mobile technology in the health care industry allows COVID-19 apps to be leveraged for risk assessment, self-management of symptoms, home monitoring, contact tracing, information sharing, training, and decision-making [8]. In this study, we examined the perceptions that may impact using mobile apps for the abovementioned purposes.

A health risk assessment is a tool used to collect information on disease status and risk; it is preemptive and can be used to manage the spread of a disease. Risk assessments can be completed by a health care professional or patient participating in self-management [12,13]. Mobile apps can be used to self-administer risk assessments, allowing individuals to identify the magnitude of their susceptibility to COVID-19. Two groups requiring risk assessments are health care workers and the general population. Health care workers are at the highest risk of contracting the disease; therefore, monitoring apps can be an effective strategy for collecting data. Researchers have used an agile methodology to develop a mobile app that identified symptomatic team members who could have posed a risk to the entire team [14], thereby establishing an effective way to assess risk. The general population can also benefit from an app-based risk assessment instrument. Researchers have found that they could predict a user’s likelihood of COVID-19 [15] after examining the data collected from a mobile app used by approximately 2.6 million users. The result aligns with our study’s aim, as it shows that apps can improve people’s awareness of their vulnerability to COVID-19.

Another way that COVID-19–related apps are helpful is that they bridge the gap in health care resources. The pandemic exacerbated the lack of health care resources. With many health care facilities at maximum capacity and staff shortages, apps helped patients with self-management [16] by facilitating the diagnosis of mild symptoms or assisting individuals in deciding when medical intervention is necessary. A French research team developed an app to track the loss of smell, a COVID-19 symptom that an affected individual can quickly identify. The data collected from the app were used to prevent the spread of COVID-19 and predict new outbreaks [17]. Another research team identified the top 5 strongest predictors for COVID-19 infection using data from a mobile app. The predictors included chills, fever, smell loss, nausea, vomiting, and shortness of breath [18]. With these predictors identified, people can be proactive in seeking care. According to the Centers for Disease Control and Prevention (CDC), except for cases of shortness of breath, individuals can use telehealth services or over-the-counter treatments [19], thereby limiting the strain on in-person clinics, hospitals, and urgent care facilities [20]. This ability of apps to limit the strain on resources is supported by a study of 3 COVID-19 apps in Thailand showing that the apps helped expand the reach of health care resources and improved the community’s health [21].

COVID-19 tracing app use can be affected by an individual’s ability to use apps effectively. The key factors that impact efficacy are app design and usability, wherein the elements can impact use and reduce the desired benefits of implementation. Research on the usability and inclusivity of COVID-19 mobile apps found that the grade level for readability exceeded the US national average. The study also found that most apps were developed for English speakers, and only a fraction of the features represented a broad cross-section of users [22]. An individual’s inability to understand instructions in an app due to high readability levels or limited language options may impact efficacy and decrease COVID-19 app use. Unfortunately, digital health equity issues, such as those mentioned above, have limited the treatment options for many vulnerable populations during the pandemic [23]. Few studies have examined digital health equity characteristics that may promote using tools like mobile apps [24]. Usability and inclusivity are salient factors that should be discussed in future studies, as feature selection may affect the use of mobile apps for infectious disease mitigation.

In a study of 12 apps used during the pandemic, including Mawid, Tabaud, Tawakkalna, Sehha, Aarogya Setu, TraceTogether, COVID safe, Immuni, COVID symptom study, COVID watch, NHS COVID-19, and PathCheck, the following features were identified: health tools, learning options, communication tools, networking tools, and safety and security options. Of note was the lack of built-in social media features in many apps [25]. In our society, where it is customary to share one’s day-to-day activities, it is unexpected that more developers would not include these features. If implemented, one use of the social media feature could be contact tracing, which identifies individuals who have been near someone newly diagnosed with COVID-19. Research shows that contact tracing apps are practical, and people will download them [26]. People must be willing to share their information for contact tracing to work. In a nationally representative survey of chronically ill individuals, only 21.8% of respondents were highly likely to share their information on a COVID-19 mobile app [27]. The authors did not examine why less than 50% of all the respondents would share their data with the mobile app developers, but it may be associated with privacy concerns.

Although addressing user concerns is essential, researchers examining data protection in contact tracing mobile apps found a need to balance protecting the society and the rights of individual patients to privacy. The government’s efforts to protect the society are substantial, as a review of 115 mobile apps showed that government agencies created the majority of apps [28]. Another study of 63 mobile apps found that 39% (the highest percentage) were developed by federal agencies [29]. Interestingly, government agencies created most apps, although private sector organizations are usually the first movers on new initiatives. Does the fact that government agencies create most COVID-19 apps impact privacy concerns? Our study is interested in information sharing; the results may elucidate why people are hesitant about sharing their data with public health officials.

With the distributed development of mobile apps across Android and iOS platforms, more individuals have access to apps; therefore, it may be easier for health care providers to implement COVID-19 tracking apps based on an individual’s preferences. Studies have shown that apps have helped people engage in self-monitoring; we will examine if patients are more likely to use COVID-19 apps if they can do so effectively. It is also evident from the studies reviewed that developers have leveraged multiple features to fight COVID-19. One of our goals was to assess whether an individual’s evaluation of risk and vulnerability to COVID-19 might induce the use of these apps. The developer’s exclusion of features that may impact privacy is noteworthy, highlighting the reason we aimed to evaluate the impact of privacy concerns on app use.

### Willingness to Use Mobile Technology

User willingness to use mobile technology has been investigated in various contexts, such as lifestyle [30], learning and education [31,32], entertainment [33], financial services [34], and health care [35]. Research in the context of mobile health care technology found that, in general, users are willing to use mobile apps when they find substantial benefits, even when they have concerns. For example, Ahadzadeh et al [36] found that patients with chronic diseases continue to use mobile health services to manage their chronic conditions when they see that the apps are helpful and satisfactory. Likewise, Zhang et al [37] found that patients are willing to use diabetes management apps to manage their conditions when they find the apps beneficial. Studies focused on fitness and wearable device apps found that most users are willing to share their information with health care providers [38]. Although prior research has shown some willingness to share data, studies have yet to examine user willingness to share COVID-19–related data. Most studies examined apps that users may see as personally beneficial. Evaluating the willingness to use COVID-19 tracing apps is critical since it also has public health ramifications.

### Theoretical Foundation and Hypothesis Development

#### PMT

Rogers posited the PMT; it is used to examine situations where individuals try to cope with or avoid noxious events to reduce a perceived threat [39]. COVID-19 is a global threat; individuals must find mechanisms to face this peril. Researchers have adapted the PMT in different contexts to evaluate how people are motivated to perform a specific behavior for hazard reduction. The use of data collection and dissemination as a method for reducing the risks of COVID-19 is appropriately evaluated using the PMT as it requires determining why people would be motivated to use the underlying mobile apps. Similarly, the PMT is used to examine protective health behaviors related to several other diseases, including schistosomiasis, HIV, and cancer, and prevention/detection behaviors, such as dieting, tobacco cessation, and exercise [40-43].

**Figure 1:**
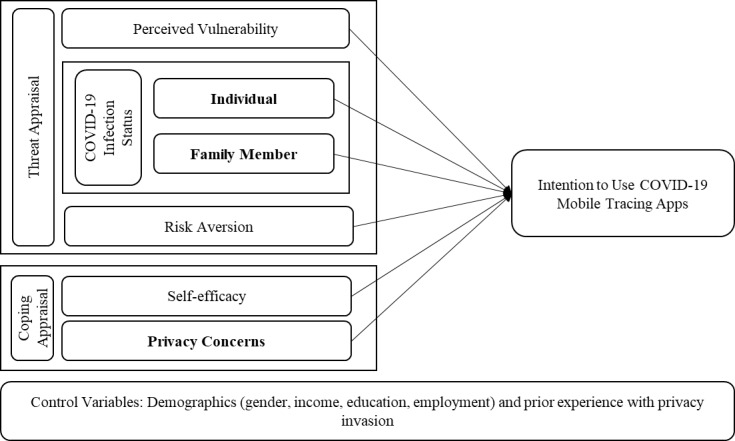
Modified protection motivation theory model. Bold text indicates modifications to the original model.

Protection motivation has 3 central stimuli: the event’s magnitude, the probability, and the availability and effectiveness of a copying response [44]. In the seminal PMT model, Rogers equated the event’s magnitude to perceived severity, the probability to a perceived vulnerability, and the response mechanism to perceived efficacy. These stimuli can be classified into threat appraisal or copying pathways. The threat appraisal pathway includes severity and vulnerability, and the copying pathway includes perceived self-efficacy and perceived cost [41]. Figure 1 depicts this study’s modified PMT model; it includes the new variables of COVID-19 infection status and risk aversion that determine a person’s perception of vulnerability in the threat appraisal pathway. The figure also illustrates the adaptation of the model to include privacy concerns as a perceived cost in the coping appraisal pathway.

#### Threat Appraisal Pathway

The threat appraisal pathway evaluates a person’s perception of a threat [41]. This appraisal is measured by the person’s belief that a disease is a threat in health care. To assess people’s threat levels, we assessed perceived vulnerability, infection status, and risk aversion. Table 1 presents a summary of the definitions of the latent variables.

**Table 1:** Summary of variable definitions.

Variable	Definition
Perceived vulnerability	Perceived vulnerability assesses how personally susceptible an individual feels to the communicated threat. Do individuals feel prone to contract COVID-19?
Risk aversion	Risk aversion refers to an individual’s reluctance to take risks and accept potential losses unless significant rewards compensate for this. In this study, risk aversion relates to the potential loss of information control by sharing data in the COVID-19 app.
Self-efficacy	Self-efficacy concerns an individual’s beliefs about whether he or she can perform the recommended coping response (related to COVID-19).
Privacy concern	Privacy concern is the apprehension over the loss of privacy and the need for protection against unwarranted communication and use of personal information. Will the wrong people gain access to my COVID-19 data?

##### Perceived Vulnerability

Perceived vulnerability assesses how personally susceptible an individual feels to the communicated threat [44]. Do individuals feel prone to contract COVID-19? In a study of Belgian nationals, researchers found that individuals who were unable to telecommute, elderly individuals, female individuals, and those with lower educational attainment felt more susceptive to COVID-19 [45]. These results are supported by the infection rates and deaths nationally and internationally. A German study characterized perceived vulnerability to COVID-19 on a continuum from high to low. Researchers found that participants across all the groups exhibited similar behaviors. Those who perceived themselves as vulnerable were more likely to practice preventive adaptive behaviors and less likely to practice risky behaviors [46]. Individuals who perceive themselves as susceptible have also shown positive protective responses to increases in the number of cases in their communities [47]. We hypothesized that perceive vulnerability will positively affect an individual’s likelihood of using a COVID-19 monitoring app (hypothesis 1).

##### COVID-19 Infection of the Individual or Family

COVID-19 infection is considered a positive test result in the polymerase chain reaction (PCR) test or an in-home rapid test [48]. This study measured an individual’s infection or the infection of a family member. This operationalization reflects the societal impact of COVID-19. People are fatigued and will likely experience psychological and emotional strain if their family members are affected [45]. Some individuals may comply with COVID-19 protocols to protect themselves or their families owing to positive COVID-19 test results. We hypothesized that an individual’s infection with COVID-19 will positively affect their likelihood of using a COVID-19 monitoring app (hypothesis 2a) and that a family member’s infection with COVID-19 will affect their likelihood of using a COVID-19 monitoring app (hypothesis 2b).

##### Risk Aversion

Risk aversion is studied extensively in health. It refers to individuals’ reluctance to take risks and accept potential losses unless significant rewards compensate for this. In our study, risk aversion was related to the potential loss of information control by sharing data in the COVID-19 app; we surmise this is a threat appraisal. One common form of risk aversion is health insurance to mitigate the risk of high costs from an unforeseen illness [49,50]. Depending on a patient’s risk aversion, they may choose plans with high or low deductibles. For example, healthy individuals (low risk aversion) choose plans with high deductibles. Similarly, individuals with high risk tolerance are found to participate in less preventative and detective behaviors, and participate in risky behaviors like lack of exercise, smoking, or alcohol abuse [51,52]. We hypothesized that risk aversion will negatively affect an individual’s likelihood of using a COVID-19 monitoring app (hypothesis 3).

#### Coping Appraisal Pathway

The coping appraisal pathway evaluates a person’s ability to cope with a threat [41]. To assess people’s ability to manage threats, we assessed self-efficacy and response cost/privacy concerns.

##### Self-efficacy

Self-efficacy concerns an individual’s beliefs about whether he or she can perform the recommended coping response [44]. To help individuals cope with COVID-19, we proposed using a mobile tracing app to better face the pandemic. One study evaluated both the technological and health care impact of self-efficacy; to do so, the researchers developed a new construct called health care technology self-efficacy (HTSE). The assessment indicated that HTSE positively influenced the attitude toward using health technologies [53]. We hypothesized that perceived self-efficacy will positively affect an individual’s likelihood of using a COVID-19 monitoring app (hypothesis 4).

##### Response Costs: Privacy Concerns

Response costs concern beliefs about how costly performing the recommended response will be to the individual [44]. Response costs can include the money, time, or effort associated with taking the adaptive coping response [54]. If I adopt the COVID-19 monitoring app, how would it impact me? Privacy concern is the apprehension over the loss of privacy and the need for protection against unwarranted communication and use of personal information [55]. Will the wrong people gain access to my COVID-19 data? Loss of privacy may be considered a response cost for using a COVID-19 monitoring app. In general, concerns over the security and privacy of protected health information have significantly impacted whether patients disclose medical information [56]. More specifically, researchers have found that patients struggle with adopting new technology due to privacy concerns [56-58]. We hypothesized that privacy concerns will negatively affect an individual’s likelihood of using a COVID-19 monitoring app (hypothesis 5).

## Methods

### Ethical Considerations

We initially applied to the institutional review board (IRB) for approval. Participants aged at least 18 years were considered for inclusion, and the population included all genders. We then collected data after receiving IRB approval (approval number: 1764316-1). The first page of the survey included the consent to participate in the study. Participants were informed that their participation is voluntary and that they may withdraw from participation at any time without adverse consequences. If they wish to withdraw, they can simply discard the survey. The form also highlighted that the survey was anonymous and participants cannot be identified. Participation in the study was completely voluntary. The following statement was present: “By taking this survey, you are consenting to participation.” Participants who completed the survey received US $1 in compensation.

### Data Collection and Summary

We collected data from 367 participants through Amazon Mechanical Turk (MTurk). Many studies have used MTurk for data collection in the health care domain [59,60]. MTurk is an online platform that connects requesters and workers (MTurk users). The features of MTurk include data collection and survey distribution. Of the 367 participants, 5 had missing responses and were removed from the data set. In addition, 37 participants did not pass the quality check question and were also removed from the data set. The final data set contained 325 valid responses from MTurk users. Of the 325 final participants, 216 were male and 109 were female. Additionally, 236 participants were employed and 89 were unemployed. Most participants (n=271) had a 4-year college degree.

Participants who chose to participate in the study were redirected to a Qualtrics link. The survey questions and responses were collected through Qualtrics. After participants finished the survey, they were required to copy a personalized survey code and input it into the MTurk survey code textbox. The first page of the survey was the “consent to participate in research” form; it explained (among other things) that participation was voluntary. The survey included multiple latent variables (privacy concerns, risk aversion, perceived vulnerability, self-efficacy, and prior privacy invasion) and some categorical variables, such as gender, education, and income. The dependent variable in the study was the intention to use a contact tracing mobile app. We also measured the participants’ experience with the disease using the following binary variables: COVID-19 infected (1=the participant has been infected with COVID-19) and COVID-19 infected-family (1=a family member has been infected with COVID-19). Table 2 shows the average Likert scale response for each latent variable.

To maintain the quality of the responses, we added a quality check question in the middle of the survey. The item asked participants to “please choose option number 3 (neither agree nor disagree).” Participants who did not select option 3 were eliminated from the study because they were not reading the questions carefully.

**Table 2:** Descriptive statistics of latent variables.

Variable	Minimum score	Maximum score	Mean score
Intention to use a contact tracing mobile app	1.00	5.00	3.8810
Privacy concerns	1.00	5.00	3.9754
Risk aversion	1.00	5.00	3.7685
Self-efficacy	1.00	5.00	4.0708
Perceived vulnerability	1.00	5.00	3.6031
Prior privacy invasion	1.00	5.00	3.3190

### Study Objective

This study assessed whether individuals would use a mobile app to give others access to data on their COVID-19 status, including infection, testing, and vaccination status. We characterized 5 constructs into 2 mediational processes to elucidate the factors that change behavioral intentions. Following the model by Roger [39], the threat appraisal pathway included perceived vulnerability. In our model, we contextualized threat appraisal by assessing the impact of COVID-19 infection on both the individual and their family. Further, to evaluate one’s sensitivity to how threatened they feel, we measured their risk aversion. We used 2 constructs to measure the coping appraisal pathway (perceived efficacy and perceived cost). Because this study assessed the use of a mobile app, we operationalized perceived cost in terms of privacy concerns. The control variables included gender, income, education, employment, and prior experience with privacy invasion.

## Results

### Measurement Model

We cleaned the data using SAS Enterprise Guide version 8.1 (SAS Institute Inc). Then, we exported the data and analyzed the measurement model using IBM SPSS AMOS version 27 (IBM Corp). Table 3 shows the results of the measurement model. The factor loading for all items was significant. The factor loading ranged from 0.588 (the lowest) to 0.923 (the highest). The results also showed that latent variables were reliable [61]. The construct reliability for the latent variables ranged from 0.754 (the lowest) to 0.914 (the highest). In addition, all latent variables satisfied the validity assessment. First, the average variance extracted (AVE) was above 0.5 for all factors (above the minimum cutoff). Moreover, the AVE scores for all factors were above the squared multiple correlations with other factors (Table 4). Finally, the overall measurement model met the guideline for a good fit model (comparative fit index=0.933, Tucker-Lewis index=0.918, root mean square error of approximation=0.065, and χ^2^/df=2.387).

**Table 3:** Measurement model results.

Variable and item		Estimate	CR^a^				AVE^b^	VIF^c^
**INT^d^**			0.914				0.780	N/A^e^
	INT_1	0.923						
	INT_2	0.864						
	INT_3	0.861						
**PC^f^**			0.826				0.544	1.348
	PC_1	0.756						
	PC_2	0.656						
	PC_3	0.737						
	PC_4	0.794						
**PPI^g^**					0.855		0.663	1.430
	PPI_1	0.841						
	PPI_2	0.790						
	PPI_3	0.811						
**PVU^h^**					0.819		0.603	1.475
	PVU_1	0.783						
	PVU_2	0.817						
	PVU_3	0.726						
**RA^i^**					0.819		0.531	1.511
	RA_1	0.690						
	RA_2	0.692						
	RA_3	0.756						
	RA_4	0.774						
**SE^j^**					0.754		0.509	1.137
	SE_1	0.744						
	SE_2	0.588						
	SE_3	0.792						

^a^CR: construct reliability.

^b^AVE: average variance extracted.

^c^VIF: variance inflation factor.

^d^INT: intention to use a contact tracing mobile app.

^e^N/A: not applicable.

^f^PC: privacy concerns.

^g^PPI: prior privacy invasion.

^h^PVU: perceived vulnerability.

^i^RA: risk aversion.

^j^SE: self-efficacy.

**Table 4:** Squared multiple correlations and average variance extracted.

Variable	INT^a^	PC^b^	PPI^c^	PVU^d^	RA^e^	SE^f^
INT	0.780^g^	0.014	0.045	0.419	0.048	0.094
PC	0.014	0.544^g^	0.223	0.086	0.214	0.071
PPI	0.045	0.223	0.663^g^	0.281	0.161	0.011
PVU	0.419	0.086	0.281	0.603^g^	0.353	0.053
RA	0.048	0.214	0.161	0.353	0.531^g^	0.135
SE	0.094	0.071	0.011	0.053	0.135	0.509^g^

^a^INT: intention to use a contact tracing mobile app.

^b^PC: privacy concerns.

^c^PPI: prior privacy invasion.

^d^PVU: perceived vulnerability.

^e^RA: risk aversion.

^f^SE: self-efficacy.

^g^Numbers in the diagonal are average variance extracted.

### Conceptual Model Results

We analyzed the theoretical model using structural equation modeling (SEM). We used AMOS version 27 (IBM Corp) to run the analysis. Table 5 shows the SEM results of the theoretical model. The results showed good model fit indices (comparative fit index=0.939, Tucker-Lewis index=0.917, root mean square error of approximation=0.054, and χ^2^/df=1.935).

First, perceived vulnerability was hypothesized to have a positive influence on the intention to use mobile tracing apps. The results also provide support. This variable showed the highest impact compared to any other variable in the model, and the impact was almost twice the impact of the second highest variable (privacy concerns). This shows that people feel vulnerable to getting infected when in close contact with others. Individuals may feel they have control over what preventive measures they take to avoid getting infected; however, they have no control over what others do. Thus, vulnerability is a big concern for individuals. Therefore, using a mobile tracing app can help people mitigate this vulnerability and increase their sense of security.

Second, we hypothesized that prior experience with the disease (COVID-19 infection of the individual or family) would show a significant influence on the intention to use a mobile tracing app. However, prior infection of a family member showed a negative impact, while prior infection of the participant showed a positive impact. This is an interesting result that perhaps needs further investigation.

**Table 5:** Structural equation modeling results.

Variable			Estimate		*P* value
**Effect**					
	INT^a^ → Perceived vulnerability	0.688		<.001	
	INT → COVID infection of the individual	0.194		.002	
	INT → COVID infection of the family	−0.139		.02	
	INT → Risk aversion	−0.150		.09	
	INT → Self-efficacy	0.292		<.001	
	INT → Privacy concerns	−0.360		<.001	
**Control variables**					
	INT → Prior privacy invasion	−0.034		.61	
	INT → Male	−0.010		.83	
	INT → Income	0.005		.91	
	INT → Employed	0.015		.75	
	INT → College degree	0.126		.04	

^a^INT: intention to use a contact tracing mobile app.

Third, we argued that risk aversion would have a negative effect on the intention to use mobile tracing apps. This hypothesis was marginally significant, with a *P* value of .09 and an estimate of −0.15. Thus, the negative influence of risk aversion and privacy concerns on sharing shows that individuals seek to avoid risk when it comes to information sharing and privacy-related issues.

Fourth, we hypothesized that self-efficacy would positively influence the intention to use mobile tracing apps. This hypothesis is also supported by the results shown in Table 5. The estimate of self-efficacy was positive at 0.292, and the *P* value was significant at <.001. This indicates that people feel confident that they can take self-preventive measures to avoid getting infected by COVID-19.

Finally, we hypothesized that privacy concerns would negatively influence the intention to use mobile tracing apps. The results of the SEM model support hypothesis 5 as the estimate was negative and significant (−0.36; *P*<.001). Thus, privacy concerns continue to be a barrier to using technology to protect the health of individuals and the public. Even with a pandemic, privacy is still important to individuals.

## Discussion

### Principal Findings

Technology is integral to patient care, as it gives health care professionals an increased capacity to communicate with patients, collect data, and diagnose and treat illnesses. Unfortunately, these benefits cannot be realized without patient adoption and the use of technology. The objective of this study was to explicate the factors that may limit or improve the adoption of technology to aid in the fight against COVID-19. This study examined individuals’ perceptions about using mobile apps that gather and monitor COVID-19–related information. The PMT was used to assess how user perception can help app development, improve adoption, and foster the use of mobile tracing apps. The results showed that an individual’s perceived vulnerability, self-efficacy, and infection with COVID-19 positively impacted the willingness to share information. Conversely, factors that negatively impacted the intention to share data on tracing apps included privacy concerns and risk aversion.

This study determined that perceived vulnerability had the highest positive impact on a person’s likelihood of using a mobile app that tracks COVID-19–related data like testing frequency, diagnosis, and vaccination status. This finding suggests that providing people with methods for assessing vulnerability may improve the adoption of mobile apps. Therefore, it is imperative to provide people with accurate information as early as possible, as prior research shows that perceived vulnerability could be manipulated based on the information people consume [46,47,62]. The knowledge gained from this study can enhance future pandemic preparation. For example, implementing tracing apps can aid individuals in determining their level of vulnerability because they can monitor infections among close contacts and use geomapping to view and possibly avoid locations with high infection rates. With the knowledge gained, consumers should be informed of potential vulnerabilities early to adopt mitigation techniques.

Efforts to implement mobile tracing apps should have specialized messaging for people who are perceived as vulnerable. Researchers at Kaiser Permanente, for example, have gathered and analyzed a wealth of data about vulnerable people; developers and implementation specialists should carefully consider the characteristics of users when creating the app and marketing material. For instance, the vulnerable population may include elderly people; therefore, the design should consider suitable features for this demographic. Organizations may consider marketing tracing apps as a way to enhance self-efficacy and assure consumers that use will not increase the risk of uncertain outcomes. Social media, videos, or even testing influencer marketing techniques can be used to achieve this aim.

In terms of self-efficacy, the results indicated that an individual’s ability to control exposure to the disease positively impacts the individual’s likelihood of using a COVID-19 tracing app. Respondents who felt they had control over activities that would expose them to COVID-19 were likely to use a mobile app. This confirms earlier findings of a positive relationship between task execution and the user’s self-efficacy [63,64]. A future study could analyze whether mitigation techniques, such as mask-wearing, social distancing, the closure of recreational facilities, and remote work significantly impact people’s perceptions of self-efficacy. This finding has implications for health care professionals and developers. Health care professionals and mobile app developers can increase the use of tracing apps and the likelihood of sharing data by providing interventions that improve app users’ self-efficacy.

Another factor with a positive impact was whether the participant had a college degree. Education was one of the control variables in our study; however, it is essential to discuss it since COVID-19 impacts the entire population. Only 42% of Americans have a college degree, so this positive finding may indicate a lower number of people who want to use tracking apps. It is not surprising since prior research has highlighted a positive relationship between patient technology use and education [65]. To ensure the equitable use of tracking apps, developers can adjust the content in the apps to meet the literacy needs of most users by applying standardized measures like the Flesch Kincaid to approximate the educational level a person requires to read the content.

The results also showed that individuals with COVID-19 infection were more likely to use mobile tracing apps. Specifically, people who contracted the disease were more willing to share data. The increased likelihood may be attributed to the experiences gained from the infection, for example, not wanting others to experience the same thing or wanting to reduce reinfection. A remarkable finding was that a family member’s infection status negatively impacted a person’s intention to use a mobile tracing app. Participants in the study may have had negative feelings about such an app since they may have believed it is too late to help a family member, that is, use would not be beneficial. Additional research is required to understand this finding fully. For example, researchers may collect data on the culture, structure, and composition of a person’s household or the type of relationship with a family member.

As hypothesized, people are less inclined to use mobile apps if they are concerned about the privacy of their information. The perception of intentional or unintentional disclosure of information to unauthorized actors may also lessen the likelihood of using tracing apps. The reasons for privacy concerns may vary; one factor may be the growing mistrust in established bodies, such as the CDC, which may monitor or collect COVID-19 data [66]. Concerns may also stem from perceptions that others could use the COVID-19 status to harm or discriminate. Further, COVID-19 has been highly politicized; therefore, privacy concerns may arise if an individual does not support the current government [67].

A multitiered governmental approach is required to protect patient data and reduce privacy concerns. In the United States, the primary regulation governing the use of patient data is the Health Insurance Portability and Accountability Act (HIPAA). Unfortunately, it is limited in its scope to protect patients’ rights to their data [68]. Attempts have been made at the federal level to improve patient protections with The 2020 Cures Act Final Rule; however, it is still limited. A more robust rule, for example, the California Consumer Privacy Act of 2018 (CCPA), provides greater protection to information. The CCPA gives consumers more control over the personal information that businesses collect about them, giving them the right to opt-out, the right to know, the right to delete, and the right to nondiscrimination. This policy is similar to the General Data Protection Regulation (GDPR) in the European Union for data protection and privacy [69]. If federation legislation can be enacted similar to the CCPA or GDPR and patients are informed of its implementation, it may go a long way in reducing privacy concerns. There are caveats to enacting new legislation alone, as studies have shown that lack of regulatory clarity and sophisticated digital infrastructure can impede the likelihood of enforcing these rules [70].

Risk aversion was another factor that had a negative impact on the likelihood of using mobile tracing apps, as indicated by the study results. Risk-averse individuals want a guaranteed outcome; for example, using a tracing app will prevent COVID-19. If the perceived benefit of using mobile tracing apps is no greater than other mitigation techniques like social distancing and vaccination, individuals will be less likely to adopt and use these apps. This finding is supported by prior research, which showed that risk-averse laypersons were overcautious when deciding whether they needed medical care [71]. To improve the adoption and use of tracing apps, health care professionals may consider reiterating that an app is a supplement to other mitigation methods, improving the odds of a guaranteed outcome. Risk aversion is also impacted by multiple social determinants of health, such as education and income [71,72]; therefore, developers and health care professionals should consider these factors to improve the adoption and use of tracing apps.

### Limitations

There are several limitations of this study. First, as noted throughout this research, the pandemic poses a significant public health threat; therefore, mitigation efforts should help a large cross-section of people. Respondents in this study were only from the United States. Therefore, the results can be applied to nations with well-developed health care infrastructure or countries with universal health care where tracing app development can be centralized. Conversely, the results may not be generalizable to countries with cultural nuances, limited health care infrastructure, or socioeconomic and political constraints. Second, COVID-19 knowledge, perceptions, and statistics change frequently. Although this study captured perceptions cross-sectionally, the factors influencing mobile tracing app use will change over time. Future research should explore additional factors that may improve the use of COVID-19 tracing apps. Third, the sample included many educated and employed participants, which may result in bias. Finally, the sample for this study was recruited using an online tool, which limited the population to those who have internet access and use online platforms regularly.

### Conclusion

Multiple factors positively and negatively influence the use of COVID-19 tracing apps. This research is salient as mobile apps can aid in information collection, dissemination, and analysis. As new variants of COVID-19 are identified and the likelihood of future pandemics lurks, access to credible information can allow individuals and health care officials to make quick decisions that will prevent the spread of highly contagious diseases. We found that perceived vulnerability to COVID-19 and privacy concerns were the 2 main factors that impacted the use of tracing apps. Accordingly, after identifying potential disease threats, health care officials should inform users of their vulnerability to diseases like COVID-19 by delivering fact-based content to improve the use of tracing apps. Significant work is required to implement and enforce health care laws protecting privacy at all government levels. Self-efficacy and one’s COVID-19 infection were associated with positive impacts on the use of tracing apps. Future disease control and prevention initiatives may benefit from using tracing apps to increase self-efficacy as it may influence one’s perception of their ability to prevent infection. Including features in apps that improve disease prevention and detection may influence risk-averse individuals, thereby reducing the negative influence on tracking app use.

This study makes various theoretical contributions. First, we adapted the PMT by operationalizing response costs as privacy concerns. Second, we adopted 2 new threat appraisals by examining risk aversion and assessing an individual’s or their family members’ COVID-19 infection status. Further, the practical implications inherent in this research are relevant to policymakers, health care practitioners, and developers. To improve the use of COVID-19 tracking apps, federal, state, local, and private sector agencies and businesses should collaborate, as the current approach lacks coordination.

## Abbreviations

AVE: average variance extracted
CCPA: California Consumer Privacy Act of 2018
CDC: Centers for Disease Control and Prevention
GDPR: General Data Protection Regulation
HTSE: health care technology self-efficacy
IRB: institutional review board
MTurk: Mechanical Turk
PMT: protection motivation theory
SEM: structural equation modeling

## Data Availability

The data sets generated during and/or analyzed during this study are available from the corresponding author on reasonable request.
